# Population pharmacokinetic-pharmacodynamic model of propofol in adolescents undergoing scoliosis surgery with intraoperative wake-up test: a study using Bispectral index and composite auditory evoked potentials as pharmacodynamic endpoints

**DOI:** 10.1186/s12871-019-0684-z

**Published:** 2019-01-22

**Authors:** Heleen J. Blussé van Oud-Alblas, Margreke J. E. Brill, Mariska Y. M. Peeters, Dick Tibboel, Meindert Danhof, Catherijne A. J. Knibbe

**Affiliations:** 1 Department of Anesthesiology, Erasmus Medical Center, Rotterdam, The Netherlands; 2 Department of Anesthesiology, St. Antonius Hospital, Nieuwegein, The Netherlands; 3 Division of Pharmacology, Leiden Academic Center for Drug Research, Leiden University, Leiden, The Netherlands; 4 Department of Clinical Pharmacy, St. Antonius Hospital, P.O. Box 2500, 3430 EM Nieuwegein, The Netherlands; 5 Department of Pediatric Intensive Care, Erasmus Medical Center - Sophia Children’s Hospital, Rotterdam, The Netherlands

**Keywords:** Propofol, Pharmacokinetics, Pharmacodynamics, Population PKPD modeling, Bispectral index, Auditory evoked potentials, Adolescents, Anesthetic depth

## Abstract

**Background:**

In adolescents limited data are available on the pharmacokinetics (PK) and pharmacodynamics (PD) of propofol. In this study we derived a PK-PD model for propofol in adolescents undergoing idiopathic scoliosis surgery with an intraoperative wake-up test with reinduction of anesthesia using both Bispectral Index (BIS) and composite A-line ARX index (cAAI) as endpoints.

**Methods:**

Fourteen adolescents (9.8–20.1 years) were evaluated during standardized propofol-remifentanil anesthesia for idiopathic scoliosis surgery with an intraoperative wake-up test with reinduction of anesthesia. BIS and cAAI were continuously measured and blood samples collected. A propofol PKPD model was developed using NONMEM.

**Results:**

The time courses of propofol concentrations, BIS and cAAI values during anesthesia, intra-operative wakeup and reduction of anesthesia were best described by a two-compartment PK model linked to an inhibitory sigmoidal Emax PD model. For the sigmoidal Emax model, the propofol concentration at half maximum effect (EC_50_) was 3.51 and 2.14 mg/L and Hill coefficient 1.43 and 6.85 for BIS and cAAI, respectively. The delay in PD effect in relation to plasma concentration was best described by a two compartment effect-site model with a ke_o_ of 0.102 min^− 1^, ke_12_ of 0.121 min^− 1^ and ke_21_ of 0.172 min^− 1^.

**Conclusions:**

A population PKPD model for propofol in adolescents was developed that successfully described the time course of propofol concentration, BIS and cAAI in individuals upon undergoing scoliosis surgery with intraoperative wake-up test and reinduction of anesthesia. Large differences were demonstrated between both monitors. This may imply that BIS and cAAI measure fundamentally different endpoints in the brain.

## Background

Propofol is frequently used for induction and maintenance of anesthesia in adults and children, as it has desirable pharmacokinetic and pharmacodynamic properties. While propofol pharmacokinetics (PK) are widely studied [1–5], there is a relative paucity of pharmacodynamic data. For adults, population models are available describing propofol PK PD during various types of surgery [6–8], different co-morbidities [9, 10], and for patients admitted to the ICU [7, 8]. To date, also a limited number of PKPD models in children have been published on the propofol dose-response relationship in children undergoing different types of surgery, and on the depth of sedation in non-ventilated children in the ICU [11–16]. However, even though adolescents are known to differ from both children and adults because of physiologic changes due to unique hormonal profiles and changes in body size and composition, only one PKPD model in (obese) adolescents was reported [17]. Moreover, whereas in adults different PD endpoints have been used in PKPD models, in children only Bispectral Index (BIS) and Comfort scores have been used to describe the time course of propofol effects in relation to the dose.

In this study we aimed to derive a population PK-PD model for propofol in adolescents undergoing idiopathic scoliosis surgery using two different PD endpoints. Scoliosis surgery provides a unique setting to study the PK and PD of propofol because during this type of surgery an intraoperative wake-up test is applied to reduce the risk of motor deficit or paraplegia. This wake-up test consists of waking up patients during and immediately after completion of spinal procedures after which anesthesia is reinduced to allow for completion of surgery and therefore allows for a unique combination of both awake and anesthetic observations in the same individual. As pharmacodynamics endpoints both BIS and composite A-line ARX index (cAAI) were used. Both BIS, derived from the EEG, and the cAAI, derived from auditory evoked potentials, have been evaluated as tools to measure depth of anesthesia.

## Methods

The study was approved by the Institutional Ethics Committee of the Erasmus University Medical Center, Rotterdam, The Netherlands. Written informed consent of parents and patients was obtained. Inclusion criteria included patients with American Society of Anesthesiologists physical status I or II undergoing correction of idiopathic scoliosis with an intraoperative wake-up test. Exclusion criteria were hypacusis or deafness, any neurological disease, medication affecting the central nervous system, or any contraindication to the anesthesia protocol.

### Anesthesia protocol

Patients received a standardized anesthetic regimen without premedication. Anesthesia was induced with remifentanil 1 mcg. kg ^− 1^. min^− 1^, followed by a bolus propofol (4 mg/kg), and rocuronium (0.6 mg/kg) to facilitate endotracheal intubation. The propofol bolus dose was administered over 10 s into a fast running saline infusion into an y-site close to the patient. The lungs were mechanically ventilated to normocapnia (end-tidal carbon dioxide 35–40 mmHg). After induction of anesthesia, a central venous catheter and an arterial line were inserted for invasive continuous measurement of central venous pressure, arterial blood pressure and for arterial blood sampling.

Anesthesia was maintained with propofol by continuous infusion (2–10 mg. kg ^− 1^. h^− 1^) and remifentanil (0.2–1 mcg. kg ^− 1^. min ^− 1^). The attending anesthesiologist conducted anesthesia in order to maintain heart rate and mean arterial pressure within 20% of baseline values obtained before induction of anesthesia. This anesthesiologist was blinded to BIS and cAAI values in order to prevent actual BIS or cAAI values to influence infusion rates of propofol, which is in accordance with current practice in our department. During the surgical procedure intrathecal morphine (5 mcg/kg) was administered by the surgeon for both intra- and postoperative pain treatment. For the wake-up test, propofol and remifentanil infusions were stopped. Patients were then repeatedly asked by the anesthesiologist in charge to move their fingers at least every 30 s until they responded, and thereafter to wiggle their toes. After finishing the wake-up test, patients were reanesthetized with an IV bolus of propofol (3–5 mg/kg) and maintenance of anesthesia was continued as previously described. Thirty minutes before the estimated end of surgery, 100 mcg/kg morphine was administered intravenously. At the end of surgery, propofol and remifentanil infusions were discontinued, and patients were extubated when sufficient spontaneous breathing had returned and they responded to verbal commands. Thereafter, patients were transferred to the Pediatric Surgical Intensive Care Unit. In addition to continuous BIS and cAAI monitoring, electrocardiogram, heart rate, noninvasive and invasive arterial blood pressure, central venous pressure, end tidal carbon dioxide, oxygen saturation via pulse oximetry, and rectal temperature were continuously monitored, collected at 5-s. intervals using Rugloop (Demed, Temse, Belgium), and synchronized with Labgrab software (Demed).

### Electroencephalographic and auditory evoked potential recording

BIS and cAAI were measured before induction of anesthesia until return of consciousness of the patient, defined as spontaneous eye opening of the patient. In the Pediatric Surgical Intensive Care only BIS was registered. In accordance with current practice BIS and cAAI values were not used to adjust propofol dosing regimens (see under Anesthesia protocol).

For the BIS, a four sensor BIS probe (BIS Pediatric Sensor, Covidien B.V. Zaltbommel, Netherlands) was attached to the left side of the patient’s forehead. Values were recorded with a BIS™ monitor (A-2000, version 3.2; Aspect Medical Systems, Newton, M.A.). The BIS is ranged from 99 to 0. Values above 90 indicate wakefulness and the target range for a patient under general anesthesia is 40 to 60. For the Auditory Evoked Potentials monitor/2 (Danmeter A/S, Odense, Denmark; software version 1.6), a headphone for auditory stimuli and three disposable electrodes (cAAI; Danmeter A/S, Odense, Denmark) were positioned at the mid-forehead (+), right forehead (reference), and right mastoid (−). The cAAI is ranged from 99 to 0. cAAI levels higher than 45 indicate wakefulness, whereas levels between 15 and 25 are considered to reflect surgical anesthesia.

### Blood sampling and analysis

Venous samples (1 ml) were taken before induction of anesthesia, and at approximately 15 or 30 min after the start of propofol infusion. After an arterial line was placed, arterial samples (1 ml) were taken at approximately 45 or 60, 120 or 180, 240 or 360 min after the start of the propofol infusion; at 5–10 or 10–15 min after stopping propofol infusion for the wake-up test; at the time of patient movement to a verbal command during the wake-up test; just before and 1 h after each dose adjustment; just before stopping; and 15 or 30, 45 or 60, and 120 min after the end of the infusion after surgery. The maximum amount for blood sampling was 25 ml per patient. Blood samples were collected in oxalate tubes and stored at 4 °C until analysis (within 1 month). Propofol concentrations were measured in whole blood using high-performance liquid chromatography with fluorescence detection [18]. The limit of quantification was 0.005 mg/l. Inter- and intra-assay coefficients of variation were less than 6.7 and 3.3%, over the concentration range 0.5–20 mg/L.

### Data analysis

The Non-Linear Mixed effect Modeling (NONMEM) program (Globomax LLC, Hanover, MD, version VI), with Splus (version 6.2; Insightful software, Seattle, WA) for the visualization of the data, was used for sequential population pharmacokinetic and pharmacodynamic analysis [19, 20]. Using population analysis, population mean parameters and inter- and intraindividual variability (i.e. residual error) are estimated, minimizing the objective function (− 2 log likelihood). The NONMEM option of the first-order conditional estimation (Method 1) with η-ε interaction was used. Model development was performed in four steps: (1) choice of structural pharmacokinetic or pharmacodynamic model (including inter-individual variability assessment), (2) choice of residual error model, (3) covariate analysis and (4) internal validation of the model. Discrimination between different models was made by comparison of the objective function. A value of *p* < 0.05, representing a decrease of 3.8 points in the objective function was considered statistically significant. In addition, goodness-of-fit plots, including observations versus individually predicted, observations versus population predicted, time versus weighted residuals, and population predicted versus weighted residuals were used for diagnostic purposes of both pharmacokinetic and pharmacodynamic data. Finally, the confidence interval of the parameter estimates, the correlation matrix and visual improvement of the individual time versus observation plots were used to evaluate the model.

### Covariate analysis

Covariates were plotted independently against the individual post-hoc parameter estimates and the weighted residuals of the basic model without covariates, to visualize potential relationships, as previously described [13]. Bodyweight, age, gender, and remifentanil infusion rates were tested as covariates.

Potential covariates were separately incorporated into the model and considered statistically significant if the objective function decreased 7.8 points (*P* < 0.005) and the 95% confidence interval of the additional parameter did not include 0 (assuming normal distribution). In addition, visual inspection of diagnostic plots was performed.

### Model evaluation

The internal validity of the population pharmacokinetic and pharmacodynamic models was primarily assessed by the bootstrap resampling method (repeated random sampling to produce another data set of the same size but with a different combination of individuals). Parameters obtained with the bootstrap replicates were compared to the estimates obtained from the original data set. Deviations up to 5–10% in structural parameters were accepted for the pharmacokinetic model and 15–20% for the pharmacodynamic model. Additionally, normalized prediction distribution errors (NPDE) were computed [21, 22].

### Pharmacokinetic model

A two-compartment pharmacokinetic model (NONMEM ADVAN 3 TRANS 4) was used to fit the log-transformed data, parameterized in terms of clearance (CL), central volume of distribution (V_1_), inter-compartmental clearance (Q) and peripheral volume of distribution (V_2_).

The individual value (post hoc value) of each parameter (P) of the ith subject was modeled by

1 $$ {\mathrm{P}}_i={{\mathrm{P}}_{\mathrm{tv}}}^{\ast }{\mathrm{e}}^{\upeta \mathrm{i}} $$

where P_tv_ is the mean estimated of the typical population parameter value and η_i_ is a random variable with mean zero and variance ω^2^, assuming log-normal distribution of the parameter in the population.

The intraindividual variability was best described by a proportional error model. This means for the jth observed log transformed propofol concentration of the ith individual (Y_ij_) the relation

2 $$ {\mathrm{Y}}_{\mathrm{ij}}=\log\ {\mathrm{c}}_{\mathrm{pred},\mathrm{ij}}+{\upvarepsilon}_{\mathrm{ij}} $$

where c_pred_ predicted log transformed propofol concentration and ε_ij_ is the random variable with mean zero and variance σ^2^.

### Pharmacodynamic model

The delay in BIS and cAAI values in relation to propofol concentration in the central pharmacokinetic compartment was characterized on the basis of a hypothetical effect compartment [23], which is an approach that has been applied before for propofol induced BIS values [24, 25].

**Fig. 1:**
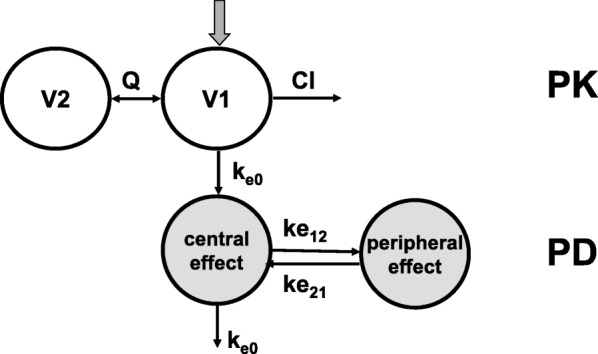
Schematic representation of the pharmacokinetic (PK) -pharmacodynamic (PD) model for propofol with V1, V2, Q and Cl representing the two compartment pharmacokinetic model and a central and peripheral effect-site compartment characterizing the pharmacodynamics. K_e0_ is the first-order rate constant from central pharmacokinetic compartment to central effect site compartment which equals the rate constant for drug loss of the effect-site compartment, k_e12_ and k_e21_ represent the rate constants from central to peripheral effect site compartment and back. The propofol concentration in the central effect site-compartment is directly responsible for the measured Bispectral Index (BIS) or composite A-Line ARX Index (cAAI) as described using eq. 4

In this effect compartment approach, it is assumed that the rate of onset and offset of effect is governed by the rate of propofol distribution to and from a hypothetical ‘effect-site’. Under this interpretation, the effect compartment is linked to the blood compartment by a first order rate constant k_e1_ and with a rate constant for drug loss k_e0_. Under the assumption that in equilibrium the effect-site concentration equals the blood concentration, the following equation can be used:

3 $$ \frac{\mathrm{dCe}}{\mathrm{dt}}\kern0.5em =\kern0.5em \mathrm{ke}0\cdot \left(\mathrm{Cb}\hbox{-} \mathrm{Ce}\right) $$

where C_b_ is the blood concentration in the central pharmacokinetic compartment, C_e_ represents the effect-site concentration and k_e0_ is the first-order equilibration constant. Beside this previously applied one compartment effect-site model for propofol, also a two compartment effect-site model was explored [26], in which distribution of propofol within the brain is represented by definition of a central effect compartment and a peripheral effect compartment (Fig. 1). Similar to the one-compartment effect model, in this two compartment effect site model, the observed effect is dependent upon the concentration in the central effect compartment, however this concentration is in its turn not only governed by the rate of propofol distribution to and from the central pharmacokinetic compartment but also by the rate of propofol distribution to and from the peripheral effect compartment, k_e12_ and k_e21_, respectively. For the pharmacodynamics model, posthoc values of the pharmacokinetic model were used. The full pharmacokinetic-pharmacodynamic model is showed in Fig. 1.

### Concentration-effect relationships

For the relation between propofol concentration in central effect compartment and BIS or cAAI values, a sigmoidal E_max_ model was used:

4 $$ {PD}_{ij}\kern0.5em =\kern0.5em {PD}_0\kern0.5em -\kern0.5em \frac{\left({E}_{\max, \kern0.5em i}\kern0.5em \bullet \kern0.5em {c}_{e, ij}^{\gamma}\right)}{{EC_{50,i}}^{\gamma}\kern0.5em +\kern0.5em {c_{e, ij}}^{\gamma }} $$

where PD_0_ is the baseline BIS or cAAI value, E_max,i_ is the maximum possible effect of propofol on BIS or cAAI in the *i*th subject, C_e,ij_ is the propofol concentration in the central effect compartment in the *i*th subject at the *j*th time point, γ is the Hill coefficient representing the steepness of the concentration versus response relation, and EC_50_ is the propofol concentration (mg/l) at half the maximum score of the BIS or cAAI. Pharmacodynamic parameters were assumed to be log-normally distributed. The interindividual variable (η_i_) was assumed to be log-normally distributed with mean zero and variance ω^2^. The residual error ε was best characterized by an additive error model.

5 $$ {Y}_{ij}\kern0.5em =\kern0.5em {PD}_{pred, ij}\kern0.5em +\kern0.5em {\varepsilon}_{ij} $$

where Y_ij_ represents the observed BIS or cAAI effect in the *i*th subject at the *j*th time point.

## Results

### Propofol pharmacokinetics

**Fig. 2:**
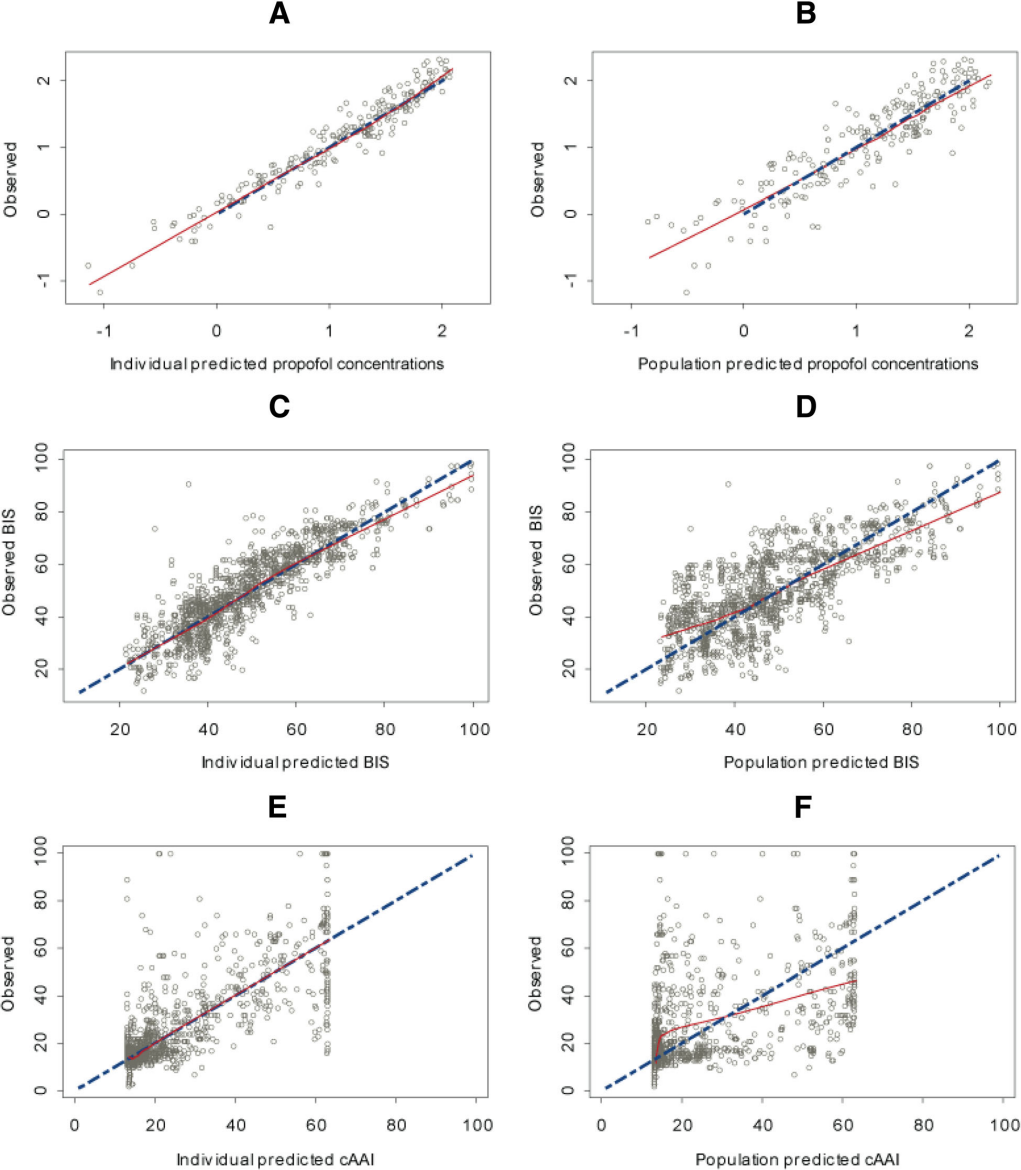
Diagnostic plots of the final pharmacokinetic (**a** and **b**) and pharmacodynamic models using Bispectral index (BIS) (**c** and **d**) and composite A-Line ARX index (cAAI) (**e** and **f**), including (left) observations versus individual-predicted propofol concentrations or depth of anesthesia, and (right) observations versus population predicted concentrations or depth of anesthesia. The solid line indicates the trend line, the dashed line represents the line of identity, x = y

**Fig. 3:**
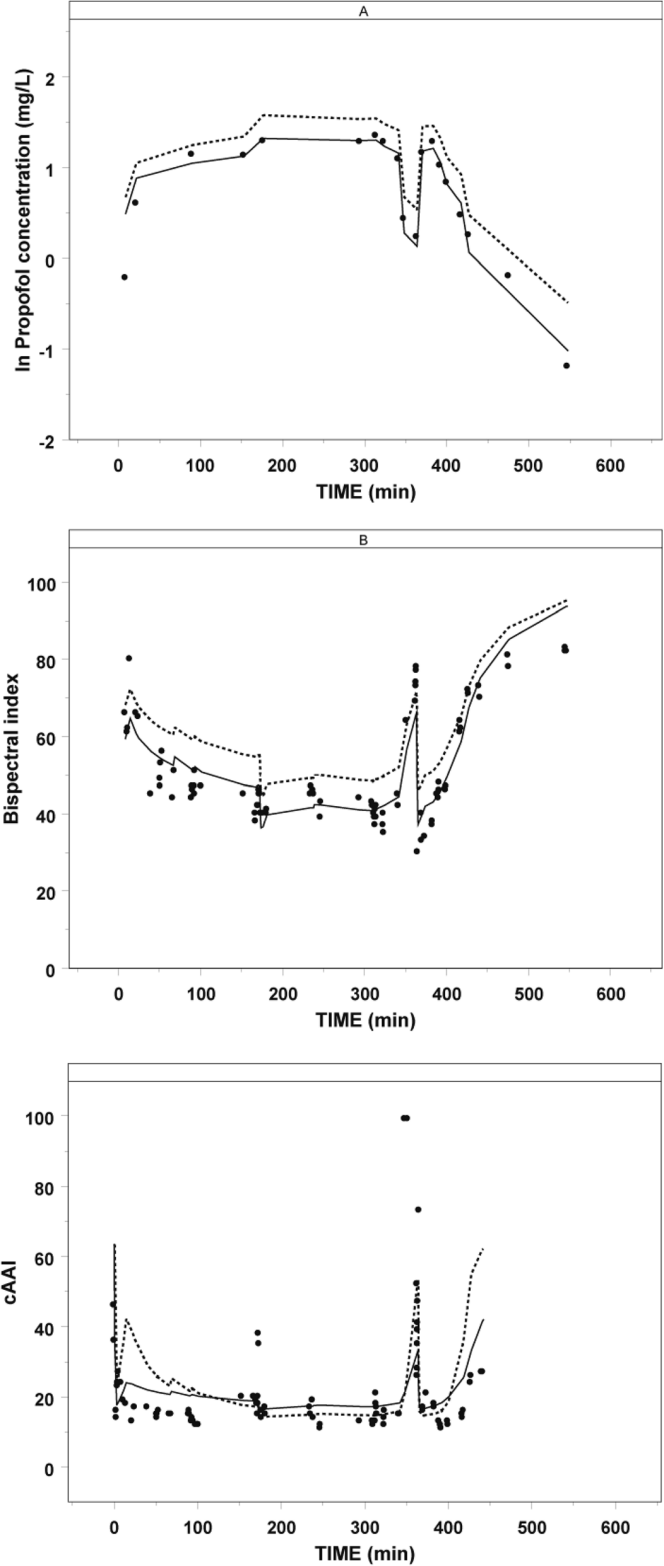
Log transformed propofol concentrations, Bispectral index and composite A-Line ARX index (cAAI) versus time (minutes) in a representative adolescent. The solid circles represent the observations, the solid lines represent the individual predicted concentration or depth of anesthesia and the dashed line represents the population predicted concentration or depth of anesthesia

The propofol pharmacokinetic model was based on 225 blood samples from 14 adolescents during propofol-remifentanil anesthesia for scoliosis surgery with an intraoperative wake-up test with reinduction of anesthesia (median duration propofol administration of 410 min). All patient and data characteristics are provided in Table 1. The pharmacokinetics of propofol were adequately described by a two-compartment model. The pharmacokinetic parameter values and their 95% confidence intervals of the final model are shown in Table 2. The fits of 1000 bootstrap replicates of the data set demonstrated the stability of the model. In Fig. 2a and b, the diagnostics of the final pharmacokinetic model is shown. Figure 3a shows the log transformed propofol concentration versus time of a representative individual. None of the explored covariates significantly influence the pharmacokinetic parameters.

**Table 1:** Patients’ and data characteristics (*n* = 14)

Gender (M / F)	2 / 12
Age (years)	14.7 (9.8–20.1)
Bodyweight (kg)	51 (36.6–82)
Height (cm)	162.5 (142–183)
Number of blood samples per patient	16 (6–22)
Number of BIS events per patient	113 (66–154)
Number of cAAI events per patient	100 (60–141)
Duration of propofol infusion (minutes)	410.0 (200–460)
Length of wake-up test (minutes)	21.5 (7.6–42.4)
ROC after propofol infusion (minutes)	52.2 (22.6–116.33)

*F* Female, *M* Male, *BIS* Bispectral Index, *cAAI* composite A-Line ARX Index, *ROC* Return of consciousness. The length of wake-up test is defined as the length of time propofol infusion was stopped during the intraoperative wake-up test during propofol-remifentanil anesthesia. Data are presented as a median (minimum-maximum)

**Table 2:** Pharmacokinetic (PK) model parameter estimates and stability of the parameters using bootstrap validation (BS)

Parameter	PK Model	BS PK Model
	Mean (CV%)	Mean (CV%)
Fixed effects		
CL (l/min)	1.37 (7.0)	1.38 (5.1)
Q (l/min)	1.15 (29.4)	1.16 (44.1)
V_1_ (l)	3.6 (10.2)	3.45 (11.3)
V_2_ (l)	76.8 (5.0)	77.3 (5.0)
Interindividual variability		
ω^2^ of CL in %	22.1 (28.7)	21.4 (25.7)
*Residual error*		
σ^2^	19.0% (11.9)	18.4% (12.5)
Performance measures		
(−2LL)	− 452	− 464.7

*BS* bootstrap validation, *CL* clearance, *Q* intercompartmental clearance, *V*_*1*_ central volume, *V*_*2*_ peripheral volume, *ω*^*2*^ variance, the square root of the exponential variance of η-1 is the percentage of the interindividual variability in the pharmacokinetic parameters; *σ*^*2*^ proportional individual variance, *-2LL* objective function. Values in parentheses are coefficient of variation (CV) of the parameter values

### Propofol pharmacodynamics

Per patient a median of 113 (66–154) observations for BIS and 100 (60–141) for cAAI were available for the pharmacodynamic analysis. Propofol concentration-effect relationship was described using a sigmoid E_max_ model (eq. 4) for which different parameter values for BIS and cAAI were found.

For BIS, the propofol concentration at half maximum effect (EC_50_) was 3.51 mg/l with interindividual coefficient of variation of 0.059 which corresponds to a CV of 25% within the context of a two compartment effect site compartment model (*p* < 0.001 compared to one-compartment effect site model). Using BIS, the delay of anesthetic effect with respect to plasma concentrations, was described by a keo of 0.102 min-1, ke12 of 0.121 min-1, ke21 of 0.172 min-1. The T_max_ of the two-compartment biophase distribution model was 2.63 min. The Hill coefficient was 1.43 (CV = 34%).

For cAAI EC50 was 2.14 mg/l (CV 42%) within the context of a one compartment effect site model with a keo of 0.067 min-1, as a two-compartment effect-site model was not superior (*P* > 0.05). For cAAI, the baseline value was estimated at 63.4 while the E_max_ was estimated at 49.8, yielding a maximum effect of 14 at the cAAI scale. The Hill coefficient was 6.85 (CV = 126%) (Table 3).

**Table 3:** Population pharmacodynamic parameters for the one-compartment effect model and two compartment effect model for propofol induced changes of the Bispectral index (BIS) and composite A-line ARX index (cAAI)

Parameter	BIS	Bs BIS	cAAI	Bs cAAI
	Mean (CV%)	Mean (%)	Mean (CV%)	Mean (%)
Fixed effects				
E_0_	100 Fixed	100 Fixed	63.4 (14.9)	63.3 (100)
E_max_ (value)	100 Fixed	100 Fixed		
E_max_ (fraction of E_0_)			0.786 (6.1)	0.787 (100)
EC_50_ (mg/l)	3.51 (6.61)	3.53 (101)	2.14 (12.4)	2.10 (98)
γ	1.43 (10.1)	1.44 (100)	6.85 (46.4)	7.03 (103)
k_eo_ (min^−1^)	0.102 (20.4)	0.103 (101)	0.067 (14.8)	0.079 (118)
k_e12_	0.121 (49.3)	0.118 (98)		
k_e21_	0.172 (35.8)	0.160 (93)		
Interindividual variability				
ω _EC50_^2^	0.059 (48.1)	0.056 (96)	0.159 (33.9)	0.165 (103)
ω γ ^2^	0.117 (41.0)	0.111 (95)	0.952 (39.3)	1.12 (118)
ω k_eo_^2^	0.192 (49.7)	0.195 (102)	0.498 (38.4)	0.522 (105)
Residual error				
σ^2^	59.5 (11.1)	58.4 (98)	133 (18.9)	122 (92)
Performance measures				
-2LL	6.984.424	6972.363 (100)	9354.40	9310.08 (100)

*BIS* Bispectral index, *cAAI* composite A-Line ARX index, *Bs* bootstrap validation (250 times), *E0* baseline, *Emax* maximum possible effect expressed as absolute value or fraction of E0, *EC50* propofol concentration at half maximum effect, *γ* Hill coefficient, *ke0* first-order equilibration constant, *ke12* rate constant from central to peripheral effect site, *ke21* rate constant from peripheral to central effect site, *ω2* variance, the square root of the exponential variance of η minus 1 is the percentage of interindividual variability in the pharmacodynamic parameters, *σ2* additive intraindividual variance, values in parentheses are *CV* coefficient of variation of the parameter values, *-2LL* objective function

For the pharmacodynamics of propofol no covariates could be identified, though there was a negatively linear trend towards an influence of age on the EC50 (*P* > 0.05) of both BIS and cAAI.

Figure 2 c, d, e, and f show diagnostic plots of the pharmacodynamic models for BIS and cAAI. In Fig. 3 b and c, BIS and cAAI values versus time in a representative adolescent are presented. These plots show that the final pharmacodynamic models describe both BIS and cAAI observations well during induction, wake-up test, reinduction and emergence of anesthesia until return of consciousness, albeit the performance was better for BIS compared to cAAI. The bootstrap validation (250 replications, Table 3) confirmed the stability of the final pharmacodynamic models. Also the results from the computation of the model NPDE demonstrated a normal distribution, with no trend in the output graphs (data not shown)

**Fig. 4:**
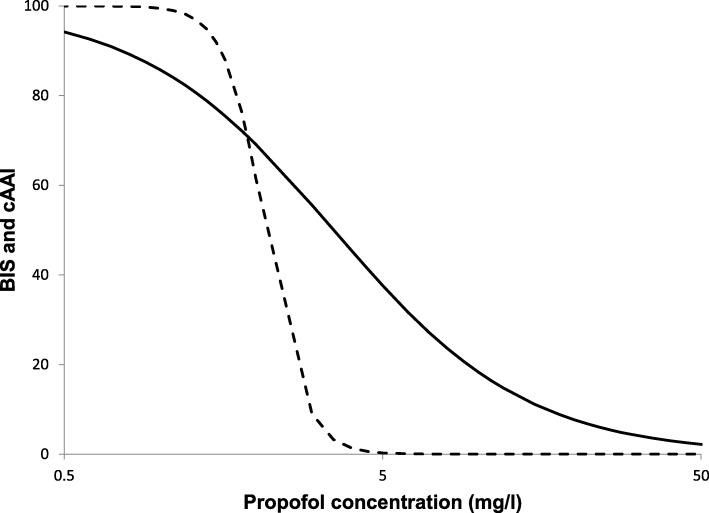
Propofol concentration – effect relation for Bispectral index (BIS, solid line) and composite A-Line ARX Index (cAAI, dotted line) for the final model

In Fig. 4 the propofol concentration effect relationships for BIS and cAAI are presented. The figure shows that BIS values gradually change as a result of the propofol concentration, while for cAAI a more rapid change in values are observed as a result of the higher Hilll coefficient.

## Discussion

In this study we derived a population PK and PD model for propofol in adolescents undergoing scoliosis surgery with an intraoperative wake-up test using BIS and cAAI as pharmacodynamic endpoints. The data used in this analysis covered a broad range of sleep and awake propofol plasma concentrations and depths of anesthesia endpoints. The time courses of propofol concentrations, BIS and cAAI values during anesthesia, intra-operative wakeup and reduction of anesthesia were best described by a two-compartment PK model linked to an inhibitory sigmoidal Emax PD model. For the sigmoidal Emax model, the propofol concentration at half maximum effect (EC50) was 3.51 and 2.14 mg/L and Hill coefficient 1.43 and 6.85 for BIS and cAAI, respectively. The delay in PD effect in relation to plasma concentration was best described by a two compartment effect-site model with a keo of 0.102 min-1, ke12 of 0.121 min-1 and ke21 of 0.172 min-1.

For the pharmacokinetics of propofol, clearance observed in this study where the median bodyweight was 51 kg, was 1.37 L/min. This result seems in accordance with the results of a recent study in (morbidly) obese adolescents and children (mean total body weight 125 kg), which demonstrated a clearance of 1.72 L/min for a 70 kg standardized individual [27]. The slightly lower propofol clearance in our (non obese) adolescents compared to that of the obese adolescents and children, may be explained by the increased blood volume and cardiac output that may be expected leading to an increased clearance in obese individuals for this high extraction ratio drug [27]. A population pharmacokinetic meta-analysis showed a clearance of 2.37 L/min with a combination of total body weight and age being the best covariates to capture changes in propofol clearance as a result of obesity and ageing which are known to influence both influence hepatic blood flow [28]. Next to this, the central volume in our study (3.6 L) is consistent with previously reported findings in adults during propofol-remifentanil anesthesia, while our estimation of the peripheral volume of distribution (76.8 L) seems very much in accordance with the earlier reported volume of 31.7 L [29].

In target-controlled-infusion (TCI) systems PK and PD models are used to determine the dose required to achieve a desired target concentration in a central or effect-site compartment. In many hospitals, propofol is administered with a target-controlled infusion (TCI) pump. Of the currently available models, the Schnider [30] and Marsh [1] pharmacokinetic models are commonly used, even though they were developed from studies in healthy subjects. Understanding the pharmacokinetics allows clinicians to adjust the delivered dose in order to control the concentration at the effect-site. Understanding the pharmacodynamics helps in titrating anesthesia according to individual needs. Simulations based on a PKPD- model will therefore lead to a propofol dosing algorithm that will result in the maintenance of adequate anesthetic depth during propofol anesthesia in each individual patient. The PKPD model that was derived in this study can be used to guide dosing for anesthesia in adolescents, which is a population that is relatively understudied. As such, the results of the current study provide an important step towards addressing propofol dosing challenges in this special population.

Our pharmacodynamic model accurately described BIS and cAAI observations during induction of anesthesia, the intraoperative wakeup test, reinduction and emergence from anesthesia (Fig. 3). Although both monitors have been proposed as pharmacodynamic endpoints, they measure the hypnotic state during anesthesia in a different way. The BIS is an empirically derived multifactorial electroencephalographic parameter that relies on the correlation of the phases between frequency components of the electroencephalogram whereas midlatency auditory evoked potentials (MLAEPS), extracted from the electroencephalogram 10–100 ms after an auditory signal, represent the earliest cortical response to an acoustic stimulus. Amplitudes and latencies of the MLAEPs are influenced by anesthetics and surgical stimuli and are therefore believed to be useful in measuring the level of hypnosis during anesthesia [31]. A monitoring variable indicating the patient’s hypnotic state, the composite A-Line ARX Index (cAAI), is then calculated from the MLAEP and the electroencephalogram. Specifically, the cAAI is preferably derived from the MLAEP, but in case of low MLAEP signal quality, it is entirely based on the spontaneous electroencephalogram. Because of these differences in measuring depth of anesthesia it is therefore not surprising that large differences were demonstrated in EC50 and Hill coefficient between both monitors implicating that both monitors measure fundamentally different endpoints in the brain.

For the BIS, depth of anesthesia was best described with a two-compartment biophase distribution model. Theoretically, this two-compartment biophase distribution model for the BIS may represent a redistribution of propofol within the central nervous system. Bjornson et al. showed that a two compartment biophase distribution best described propofol effect measured by BIS in healthy adult volunteers [26]. Earlier Upton and Ludbrook, when defining a physiological PK (PBPK) model of propofol in adult man, found a two-compartment brain model to best describe BIS observations [28].

For the cAAI, depth of sedation was best described by a classic one-compartment effect site model with a sigmodial Emax pharmacodynamic model and no propofol biophase distribution could be characterized for this measure. This finding may be explained by the very steep Hill coefficient (on-off phenomenon) together with low resolution sampling of the pharmacodynamic measurements during the rapid changes in cAAI. Interestingly, the steeper Hill coefficient for cAAI supports our clinical observation that the awaking from propofol remifentanil anesthesia was associated with an ‘on/off’ cAAI response rather than a gradual progression to awake levels. Further differences between the BIS and cAAI models suggest that they measure significantly different endpoints in the brain, which is reflected by large differences in EC50 (3.51 mg/l for the BIS versus 2.14 mg/l for the cAAI). Research into these differences is needed to elucidate the physiologic- and monitor-based rational of these results.

Although large differences between Emax models of cAAI and BIS measures are found, based on this study there is no evidence that might support the use of one or the other monitor of depth of anesthesia, BIS or cAAI. On the other hand, an instrument of which the value gradually changes with propofol concentration may be of advantage when aiming for an optimal level of anesthesia through titration, which favors the BIS compared to cAAI.

Patients included in this study were instructed to remember a color during the wake/up test, and were postoperatively interviewed for explicit recall. Results from this separate part of our study have been previously published [32]. Although all patients responded adequately to the wake/up test (e.g. by wiggling their toes or feet) and both EEG indices demonstrated patients being awake, no explicit recall or postoperative pain was demonstrated [32]. Furthermore, in this study we reported on the performance of both indices in distinguishing different hypnotic states, as evaluated by the University of Michigan Sedation Scale, during scoliosis surgery with an intraoperative wake-up test [32]. BIS and cAAI were compared at the start of the wake-up test, at purposeful movement to command, and after the patient was reanesthetized. During induction of anesthesia, BIS performed better than cAAI, as indicated by prediction probabilities. Although cAAI was statistically a better discriminator for the level of consciousness during the wake-up test and emergence, these differences do not appear to be clinically meaningful. Both indices increased during the wake-up test, indicating a higher level of consciousness. However, both monitors were not found to be ideal, as considerable overlap in both BIS and cAAI values was present when compared with each level of the UMSS [32].

No covariates could be identified for the pharmacokinetic and for the pharmacodynamic model of propofol, although there was a trend towards an influence of bodyweight on clearance as well as age on the EC50. Another study investigating propofol effects in adolescents studied propofol BIS effect in children (6–13) and adults (13–35 years) [11]. They found, that when pooling adult and children data, the fit with the Schnider model significantly improved when puberty status (child or adult) was added to the model as a covariate, showing that age probably has an influence on the effect of propofol. These results and those of the present study seem to be in line and future studies in a large age range will have to show whether age indeed is of influence for propofol pharmacodynamics. As remifentanil was co-administered with propofol in this study, its potential influence on the pharmacokinetics or pharmacodynamics of propofol was investigated, however no influence could be identified. This seems to be in contrast with literature in adults, in which synergy is reported between remifentanil and propofol, with modest remifentanil concentrations already dramatically reducing the concentrations of propofol required to ablate a response [31]. More specifically, also in children, remifentanil was found to reduce the concentration of propofol required for anesthesia but increasing the remifentanil infusion yielded minimal additional effect [33]. As all children in our study received remifentanil infusions during this prolonged surgical procedure in moderate to high dosages (2–10 mcg.kg-1.h-1), we believe that we have reached maximum synergy at the dose levels used. This may explain why we could not identify an influence of moderate versus high remifentanil infusion rates on the pharmacokinetics and pharmacodynamics of propofol in our patients.

A limitation of our study was that due to ethical considerations, propofol sampling during induction of anesthesia was limited. However, the intraoperative wake-up test during scoliosis surgery allowed for extra propofol sampling after reinduction of anesthesia. Furthermore, because of the relatively small amount of available awake data points in the present study, it was not possible to estimate baseline and Emax for BIS. Therefore, in the final pharmacodynamic model both parameters for BIS were fixed to 100. For cAAI, baseline was estimated at 63.4, and Emax at 49.8. Interestingly, fixing the Emax for cAAI to 100 gave a similar EC50 value, indicating a minimal influence on EC50. Another limitation of the final pharmacodynamic models is that intraoperative stimuli during the wake up test were not accounted for. Model based predictions following the recommended propofol dosing regimen will therefore predict cAAI and BIS values without a stimulus to move legs or arms and may therefore underestimate peak cAAI values (Fig. 3c) and perhaps also BIS values (Fig. 3b), if the wake-up test is accompanied by verbal and surgical stimulation. Finally, although age tended to influence EC50 (*p* > 0.05), no covariates of propofol could be identified in the present study. This is probably due to insufficient statistical power to estimate a continuous influence of age on EC50. Yet, when a separate EC50 for the youngest subject was estimated, a significant decrease in the NONMEM objective function was observed. Future studies in younger individuals will have to explore whether age is of influence to EC50.

## Conclusion

In conclusion, a population PKPD model for propofol in adolescents was developed that successfully described the time course and variability of propofol concentration, BIS and cAAI in individuals upon undergoing scoliosis surgery with intraoperative wake-up test and reinduction of anesthesia. Large differences were demonstrated between both monitors. This may imply that BIS and cAAI measure fundamentally different endpoints in the brain.

Comprehensive knowledge of propofol PKPD in this specific age group is of importance, as this will lead to an increased understanding of propofol effects and will eventually lead to evidence-based dosing strategies in this population.

## Abbreviations

BIS: Bispectral index
cAAI: composite A-line ARX index
NONMEM: Non-Linear Mixed effect Modeling
NPDE: normalized prediction distribution errors
PD: pharmacodynamics
PK: pharmacokinetics
